# Purification and the Secondary Structure of Fucoidanase from *Fusarium* sp. LD8

**DOI:** 10.1155/2011/196190

**Published:** 2011-10-01

**Authors:** Wu Qianqian, Ma Shuang, Xiao Hourong, Zhang Min, Cai Jingmin

**Affiliations:** Department of Biological and Environmental Sciences, Hefei University, Hefei 230022, China

## Abstract

The fucoidanase from *Fusarium* sp. (LD8) was obtained by solid-state fermentation. The fermented solid medium was extracted by citric acid buffer, and the extracts were precipitated by acetone and purified by Sephadex G-100 successively. The results showed that the specific fucoidanase activity of purified enzyme was 22.7-fold than that of the crude enzyme. The recovery of the enzyme was 23.9%. The purified enzyme gave a single band on SDS-PAGE gel, and the molecular weight of fucoidanase was about 64 kDa. The isoelectric point of the enzyme was 4.5. The enzyme properties were also studied. The results showed that the optimum temperature and pH were 60°C and 6.0, respectively; the temperature of half inactivation was 50°C, and the most stable pH for the enzyme was 6.0. *K*_*M*_, and the *V*_max_ of the enzyme was 8.9 mg**·**L^−1^ and 2.02 mg**·**min^−1^ **·**mL^−1^ by using fucoidan from *Fucus vesiculosus* as substrate. The compositions of the secondary structure of fucoidanase were estimated by FTIR, the second derivative spectra, and the curve-fitting analysis of the amide I bands in their spectra. The results showed that *β*-sheet was the dominant component (58.6%) and *α*-helix was the least (12%); the content of *β*-turn and random coil were 15.39% and 14.5%, respectively.

## 1. Introduction

Sulfated polysaccharides, named fucoidan, could be extracted from many marine brown algae. The main differences of structural characterization in algal fucoidan originate from their species; the backbone of fucoidan isolated from *Laminaria cichorioides *[1] was consisting of *α* (1–3) linked fucopyranose residues, and that from *L. japonica *was primarily *α* (1–3) linked fucopyranose residues (75%) and a few *α* (1–4) fucopyranose linkages (25%) [2]; The fucoidan from *Fucus serratus *[3],* F. evanescens *[4], and *F. distichus* [5] was shown to contain a backbone consisting of alternating (1–3) and (1–4) linked a-L-fucopyranose residues.

Investigators were interested in screening fucoidan which was isolated from different brown algal species; fucoidan was attributed several different bioactivities including anticoagulant [6–8], antithrombotic/antithrombin activity [9], antiviral [10], and other activities [11]. The biological effects of fucoidan depended on the molecular mass, sulfate content, and sugar constituents [12, 13]. The low molecular weight fucoidan (LMWF) could be obtained by fucoidanase (E.C.3.2.1.44) enzymolysis, and it could hydrolyze fucoidan to sulfated LMWF without removal of its side substitute groups. 

Fucoidanase could be extracted from hepatopancreas of invertebrates [14, 15], marine bacteria [16–25], and marine fungi [26–28].

There were some papers focusing on fermentation conditions of fucoidanase from marine fungus [26–28] and marine bacteria [23, 29], a few papers concerning purifications of fucoidanase from hepatopancreas [14] and marine bacteria reported [17, 20–22]. In order to elucidate the structure of fungal fucoidanase, we investigate fucoidanases isolated from different species of fungi. The present work is devoted to purification and the structure analysis of a fucoidanase from marine fungus *Fusarium* sp. LD8.

## 2. Materials and Methods

### 2.1. Chemicals

Fucoidan from* Fucus vesiculosus *was obtained from Sigma Chemical Co., Sephadex; G-100 was purchased from Pharmacia Biotech Corporation (Sweden). Fucoidan from* Laminaria *sp. was purchased from Rizhao Jiejing Ocean Biotechnology Development Co., Ltd (PRC). All of the other chemical reagents were analytical pure grade made in China.

### 2.2. Microorganism

The marine fungus LD8 was isolated from sand of North Sea in German which was identified as *Fusarium* sp. (LD8), and it could be used for the synthesis of fucoidanase. Solid-state medium for LD8 consisted of 7.5 g wheat bran, 0.5 g kelp powder, 0.1 g glucose, 0.04 g NaNO_3_, and 7.5 mL artificial sea water in 250 mL flasks [27].

### 2.3. Chemical Analysis

The protein concentration was determined by the Bradford method [30].

### 2.4. Enzyme Assay

#### 2.4.1. Fucoidanase Activity

Fucoidanase activity was measured by the dinitrosalicylic acid technique [31] to estimate the release of reducing sugars at 540 nm as follows: a mixture consisting of 1 mL substrate solution (1% fucoidan (w/v) from Fucus vesiculosus dissolved with 0.02 mol*·*L^−1^ citric acid-sodium citric buffer, pH 6.0) and 0.1 mL enzyme solution (crude extract or pure enzyme) was incubated at 50°C for 10 min, using inactivated enzyme solution as blank CK. Using fucose as standard, the calibration curve function was *y* = 1.611*x* (*x*: the quantity of fucose, mg; *y*: the absorbance at 540 nm, *R* ^2^ = 0.9983).

One unit (IU) of fucoidanase activity is defined as the amount of enzyme that releases 1 *μ*mol of fucose per minute under the assay conditions.

#### 2.4.2. Fucosidase Activity

Fucosidase activity was measured under the following conditions: the reaction mixture contained 1 mL substrate solution (1%  *ρ*-nitrophenyl-*α*-L-fucoside (w/v) dissolved with 0.02 mol*·*L^−1^ citric acid-sodium citric buffer, pH 6.0) and 0.1 mL enzyme solution (purified enzyme) was incubated at 40°C for 2 h. One unit (IU) of fucosidase activity is defined as the amount of enzyme that releases 1 *μ*mol of *ρ*-nitrophenyl per minute under the assay conditions.

#### 2.4.3. Amylase Assay

The reaction mixture containing 1 mL of 2% soluble starch (w/v) in acetate buffer (pH 5.5) and 1 mL of enzyme solution was incubated with shaking at 40°C for 30 min. The reaction was stopped by boiling water for 5 min, and after centrifugation the released reducing sugar was measured by the dinitrosalicylic acid method [31]. One unit (IU) of amylase activity is defined as the amount of enzyme that liberated 1 *μ*mol reducing sugar (as glucose) per min under the assay conditions.

### 2.5. Extraction, Purification, and Purity Identification of Fucoidanase

The LD8 was cultivated at 28°C for 48 h on the solid-state medium in 250 mL flask.

All the following steps were accomplished at 4°C except being indicated specifically.

50 g fermented culture medium was extracted with citric acid-citric sodium buffer (pH 6.0) for 0.5 h. After being filtrated through six-layer carbasus, the filtrate was centrifuged at 15,000 g for 0.5 h, and then ice-cold acetone was added to the supernatant to a final concentration of 66.7% (v/v) with gentle stirring. Insoluble material was obtained by centrifugation at 15,000 g for 0.5 h. The precipitate was dissolved in pH 6.0, 0.02 mol*·*L^−1^ citric acid-citric sodium buffer and was centrifuged at 15,000 g for 0.5 h, and the clear solution was collected for use. The enzyme solution was concentrated to about 10 mL by low-temperature vacuum concentration and then loaded to Sephadex G-100 column (2.5 × 100 cm), which has been balanced well with 0.1 mol*·*L^−1^ citric acid-citric sodium buffer. The enzyme was eluted at room temperature at the flow rate of 0.33 mL*·*min^−1^; fractions were collected at 20 min intervals. The fractions with the highest enzymatic activity were pooled, concentrated by low-temperature vacuum concentration to 10 mL, dialyzed in deionized water, lyophilized, and stored at −18°C.

SDS-PAGE was performed to identify the purity of the purified enzyme and calculate the *M*_*w*_ of the proteins in the gel. SDS-PAGE was 7% (w/v) polyacrylamide gel containing 10% (w/v) SDS; molecular markers were myosin (220 kDa), *α*-2 macroglobulin (170 kDa), *β*-galactosidase (116 kDa), transferrin (76 kDa), and glutamic dehydrogenase (53 kDa), respectively. The protein was stained by Coomassie bright blue R-250.

### 2.6. Characteristics of the Purified Enzyme

Isoelectrofocusing was performed on gel rods. The electrode solutions were 2% (w/v) sodium hydroxide solution at the cathode and 5% (v/v) phosphoric acid solution at the anode. Each gel was focused in 100 V, 2 mA for 2 h, followed by 150–160 V, 4 mA for 5 h, which allowed for a sharp focalisation of fucoidanase. Following isoelectric focusing, the gels were cut into 0.5 cm for measuring pH and enzyme activity. The protein was stained by 0.5% amino black (w/v).

The pH relative activity of the fucoidanase was determined by detecting the fucoidanase activity over a pH range of 3–8 with three kinds of buffer solutions (50 mmol*·*L^−1^ sodium citrate buffer, 50 mmol*·*L^−1^ sodium phosphate buffer, 50 mmol*·*L^−1^ sodium carbonate buffer) at 50°C. The pH stability of the enzyme was studied by detecting the residual activity after the enzyme being incubated for 3 h at room temperature under different pH value. To obtain optimal reactive temperature, a mixture consisting of 1 mL substrate solution (1% fucoidan (w/v) from *Fucus vesiculosus* dissolved with 0.02 mol*·*L^−1^ citric acid-sodium citric buffer, pH 6.0) and 0.1 mL enzyme solution was incubated at different temperatures for 10 min. For thermal stability study, 0.1 mL of enzyme solution was incubated at temperature range from 30 to 80°C for 1 h, then cooled rapidly in ice bath for 5 min, and then removed to 25°C. The residual enzyme activity was detected at 50°C for 10 min.

The substrate specificity of the enzyme was examined by using different fucoidan from* Fucus vesiculosus* and *Laminaria* sp. as substrates. Michaelis constants (*K*_*M*_) and maximum reaction velocities (*V*_max_) were calculated by the double-reciprocal plot method of Lineweaver and Burk [32].

### 2.7. FTIR Assessment of the Secondary Structure of Fucoidanase

Infrared spectra were recorded on a MAGNA-IR750 Fourier transform spectrometer (INCOLET INSTRVMENTW, USA). For the spectrum range from 1500~2200 cm^−1^, 32 scans were collected at a spectral resolution of 4 cm^−1^. Pure fucoidanase (10 mg) was mixed with 100 mg of dried potassium bromide (KBr). Water vapor was purged from sample room. The spectrum of the amide I band of fucoidanase was obtained, and self-deconvolution and curve-fitting methods were used to analyze the secondary structure of the fucoidanase.

## 3. Result

### 3.1. Purification of Fucoidanase

**Figure 1:**
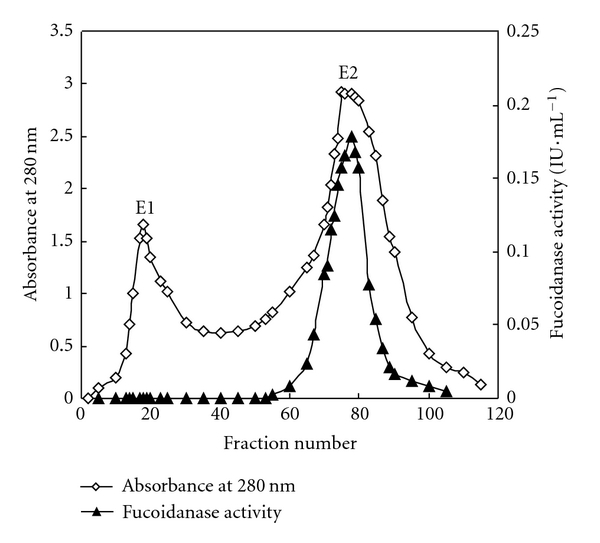
Gel filtration column chromatography. Chromatography of fucoidanase obtained from the column of Sephadex G-100 (2.5 × 100 cm). After the column had been washed well with 0.1 mol*·*L^−1^ citric acid-citric sodium buffer (pH 6.0), it was eluted with 2000 mL of the same buffer solution at a flow rate of 0.33 mL*·*min^−1^ with 6.6 mL elute in each fraction.

Two protein peaks (E1 and E2) were observed. E2 peak showed fucoidanase activities (Figure 1) whose purity had been detected by SDS-PAGE (see below). The crude enzyme extraction was purified by 66.7% acetone precipitation and Sephadex G-100 gel chromatography (Table 1). The purification fold of fucoidanase activity of 1 mg protein was enhanced from 1-fold to 23.9-folds while recovery rate was decreased from 100% to 22.7%. The fractions with higher fucoidanase activity (tube no. 65 to 87) were pooled and concentrated to 10 mL. To exclude the reducing carbohydrates from carbon sources used for cultivation (starch, kelp polysaccharides), fucoidanase-related enzymes such as fucosidase and amylase activity of purified protein were determined, but there is no fucosidase or amylase activity (Table 2).

### 3.2. Determination of Isoelectric Point and Molecular Mass of Fucoidanase

**Figure 2:**
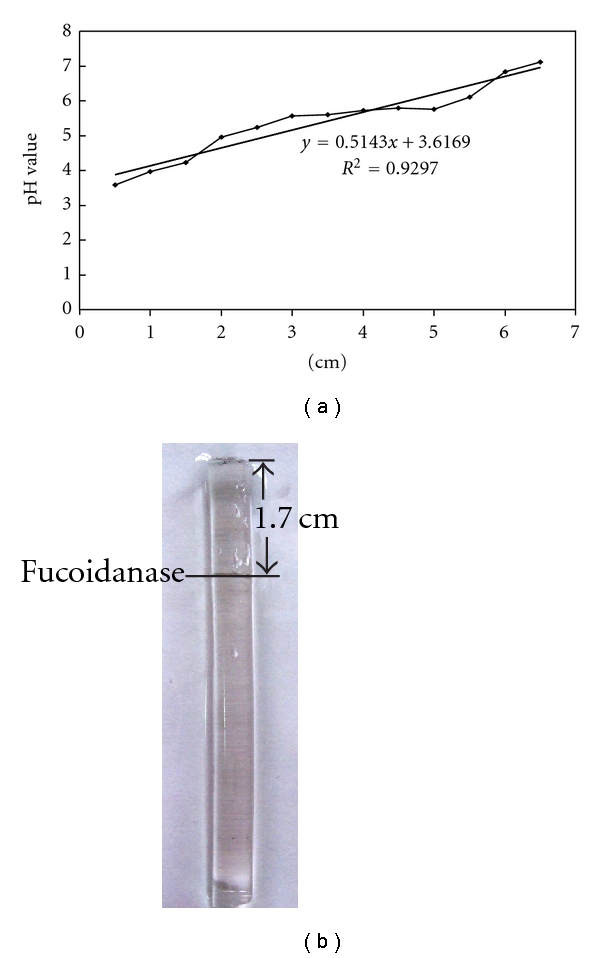
Determination of isoelectric point of the fucoidanase (a) *R*_*f*_ value of the pH; (b) *R*_*f*_ of the purified fucoidanase on original isoelectric focusing gel isoelectrofocusing was performed on gel rods. The electrode solutions were 2% (w/v) sodium hydroxide solution at the cathode and 5% (v/v) phosphoric acid solution at the anode. Each gel was focused in 100 V, 2 mA for 2 h, followed by 150–160 V, 4 mA for 5 h. Following isoelectric focusing, the gels were cut into 0.5 cm for measuring pH and enzyme activity. The protein was stained by 0.5% amino black.

The result exhibited that the purified fucoidanase gave a single band on isoelectric electrophoresis, and the isoelectric point of the enzyme was pH 4.5 (Figure 2). 

**Figure 3:**
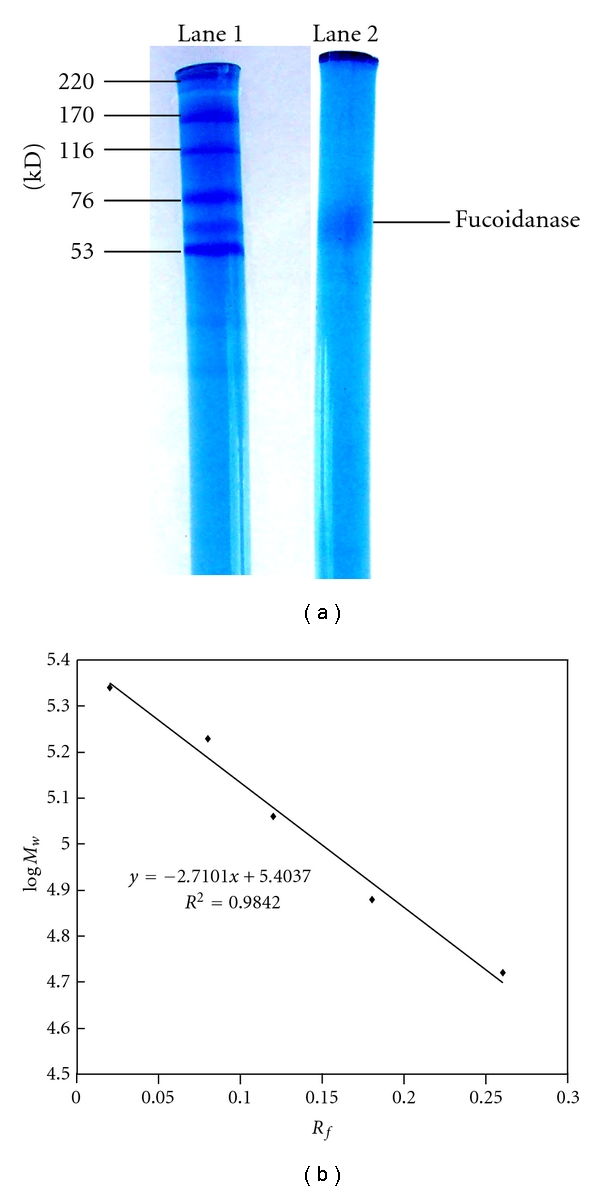
Determination of the *M*_*w*_ of the fucoidanase by SDS-PAGE. Picture of electrophoresis of fucoidanase: lane 1 is *M*_*w*_ marker; lane 2 is the fucoidanase; (b) standard curve and *M*_*w*_ function of the *M*_*w*_ marker SDS-PAGE was conducted using discontinuous electrophoresis method with Phastsystem, 5.5% (w/v) polyacrylamide, pH 8.3, 250 V, 10 mA, in the concentration gel with 7.0% polyacrylamide (w/v), 250 V, 30 mA, in the separation gel. Protein was stained with Coomassie brilliant blue R-250. Molecular markers used were (220 kDa), *α*-2 macroglobulin (170 kDa), *β*-galactosidase (116 kDa), transferring (76 kDa), and glutamic dehydrogenase (53 kDa).

The purified fucoidanase gave a single band on SDS-PAGE gel, which suggested that relatively pure fucoidanase had been obtained. The molecular mass of the fucoidanase was about 64 kDa by SDS-PAGE (Figure 3) which was different from that of* Dendryphiella arenaria* TM94 (180 kDa). Molecular markers used were myosin (220 kDa), *α*-2 macroglobulin (170 kDa), *β*-galactosidase (116 kDa), transferrin (76 kDa), and glutamic dehydrogenase (53 kDa), respectively.

### 3.3. Effect of pH on Fucoidanase Activity and Stability

**Figure 4:**
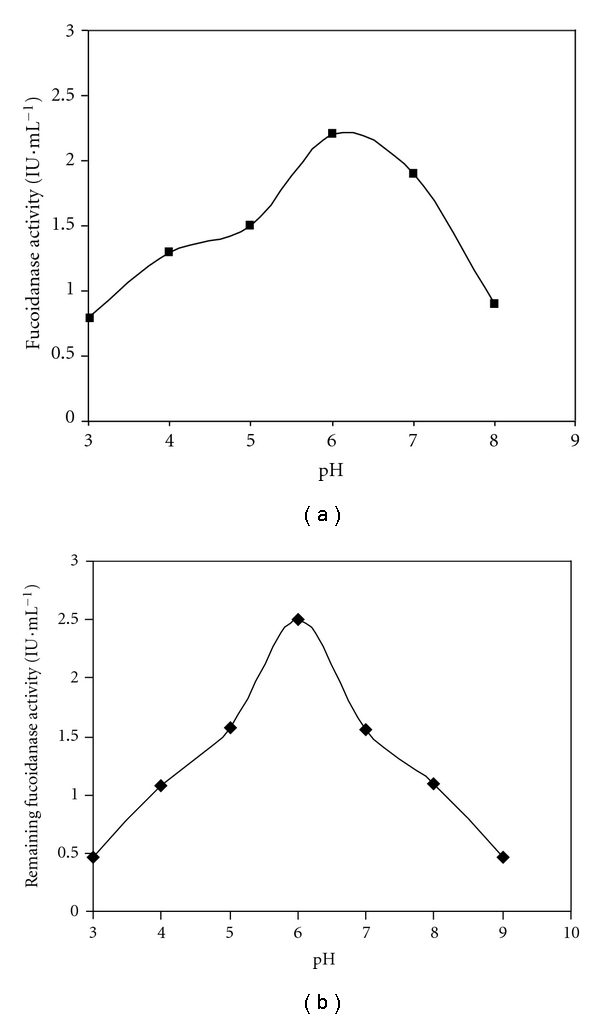
Effects of pH on fucoidanase activity (a) and stability. (b) Effects of pH on fucoidanase activity was determined under the following method: 1.0 mL substrate solution (pH 3–8) in buffer solutions (50 mmol*·*L^−1^ sodium citrate buffer, 50 mmol*·*L^−1^ sodium phosphate buffer, 50 mmol*·*L^−1^ sodium carbonate buffer) was added to 0.1 mL enzyme solution and then was incubated at 50°C for 10 min. The pH stability of the enzyme was determined by assaying the residual activity after incubating enzyme for 3 h at the room temperature at different pH levels ranging from 3 to 9. Residual fucoidanase activity was determined by adding 0.1 mL enzyme solution to 1.0 mL of substrate solution (citric acid-citric sodium buffer, pH 6.0) and then was incubated at 50°C for 10 min.

Effect of pH on the activity of fucoidanase obtained from *Fusarium *sp. LD8 was shown in Figure 4. The maximum enzyme activity was at pH 6. The optimal pH of this enzyme was very close to that from marine fungus* Dendryphiella arenaria* TM94 and *Vibrio* sp. N-5, while the optimal pH of fucoidanase from Hepatopancreas of *Patinopecten yessoensis* was 5.5.

The effect of pH on stability was also determined. The results showed that the enzyme displayed stability at pH 6.0, whereas at pH 5.0 and 8.0, an activity loss of about 50% occurred after 6 h incubation at room temperature (25°C), respectively.

### 3.4. Effect of Temperature on Fucoidanase Activity and Stability

**Figure 5:**
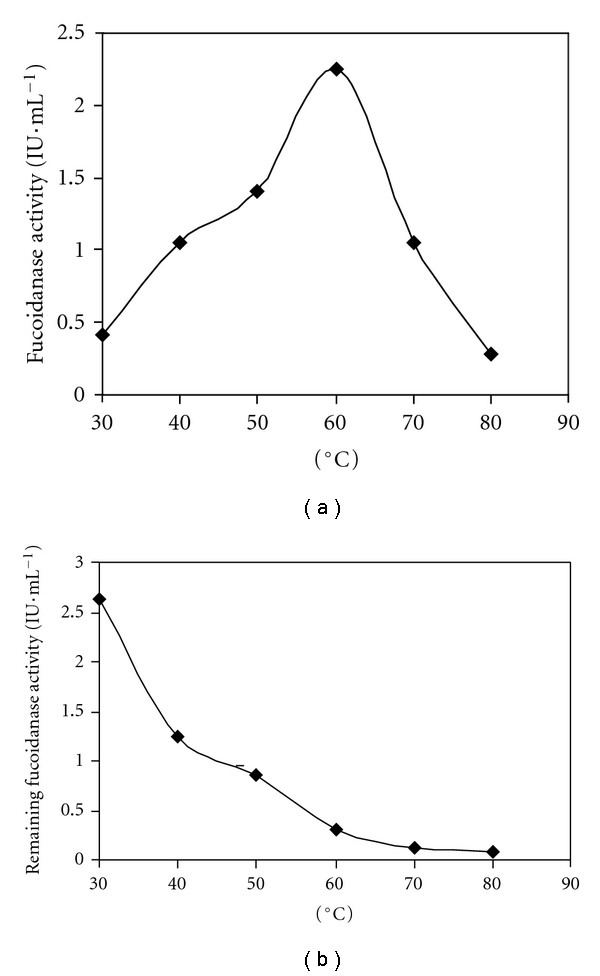
Effects of temperature on fucoidanase activity (a) and stability. (b) Effects of temperature on fucoidanase activity was obtained by the following method: adding 0.1 mL of enzyme solution to 1.0 mL of substrate solution (citric acid-citric sodium buffer, pH 6.0), then incubating for 10 min at different temperatures from 30°C to 80°C, respectively. Temperature on stability of the fucoidanase was determined by assaying the residual activity under the following method: 0.1 mL of enzyme solution was incubated at 30 to 80°C for 1 h, rapidly cooled in an ice bath for 5 min, and then removed to 25°C. When the sample reached 25°C, 1.0 mL of substrate solution was added, and the residual enzyme activity was determined and expressed relative to the maximum activity.

The optimum temperature for maximal activity of the fucoidanase was 60°C at pH 6.0 (Figure 5(a)). At 30°C the activity of fucoidanase decreased to 12.5%, while at 80°C to 18.75%. The result showed that the optimal temperature of fucoidanase from TM94 was higher than that of the fucoidanase in *Vibrio* sp. N-5, whose optimum temperature was 40°C [33].

The residual activity of fucoidanase was examined after preincubating it at different temperatures for 1 hr, and the temperature at which enzyme lost half activity was 50°C (Figure 5(b)). It was completely inactivated at above 80°C. The temperature of lost half activity is different from those of *Vibrio* sp. N-5 and *Dendryphiella arenaria* TM94. The enzyme of* Dendryphiella arenaria* TM94 and *Vibrio* sp. N-5 showed that their optimal temperature of half lost inactivation was at 56°C and 40°C, respectively [33].

### 3.5. Determination of *K*_M_, *V*_max_ and Affinity for Fucoidanase

The kinetic parameters of fucoidanase were examined using *Fucus vesiculosus* fucoidan and *Laminaria* sp. fucoidan as substrates. The *K*_*M*_ values of the enzyme determined by Lineweaver-Burk method were 8.9 mg*·*L^−1^ and 10.9 mg*·*mL^−1^, respectively. At the same time, the *V*_max_ values for both substrates were 2.02 mg*·*min^−1^ *·*mL^−1^ and 2.06 mg*·*min^−1^ *·*mL^−1^, respectively. The enzyme showed higher affinity to fucoidan from *Fucus vesiculosus.*

### 3.6. The Second Structure of Fucoidanase

**Figure 6:**
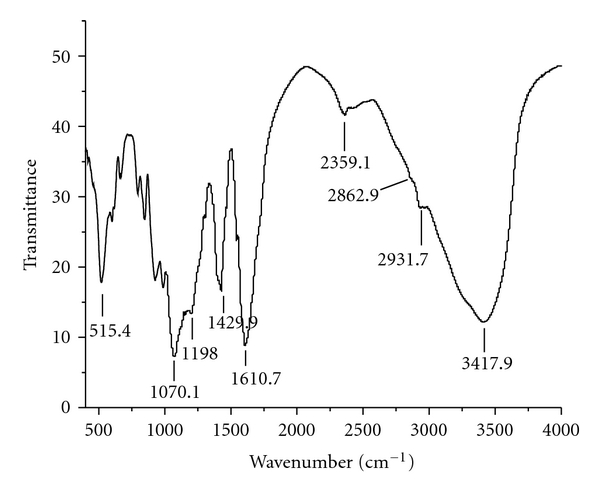
The FTIR spectra of fucoidanase. Fucoidanase (10 mg) was mixed with 100 mg of dried potassium bromide (KBr). Water vapor was purged from sample room. A baseline drawn between the spectra data points at 1500 and 2200 cm^−1^; 32 scans were collected at a spectral resolution of 4 cm^−1^.

The original IR spectrum for the fucoidanase was showed in Figure 6. The bands at about 1620~1700 cm^−1^ can be mainly attributed to the (*ν*(C=O)) and is called amide I. The shape of the amide I band is representative of fucoidanase secondary structure. The bands of the 1650~1658 cm^−1^, 1600~1640 cm^−1^, 1640~1650 cm^−1^, 1660~1695 cm^−1^ regions were, respectively, assigned to *α*-helix, *β*-sheet, random coil, and *β*-turn structure. 

The second derivative and a curve-fitting treatment can be carried out to estimate quantitatively the relative proportion of each component representing a type of secondary structure. The fourth derivative function was calculated by the PeakFit 4.12 software to determine the number of components in the amide I region for the second derivative spectra and the curve-fitting process (Table 3). According to this band composition, the amide I profile of fucoidanase contains four major components that can be linked with *α*-helix, *β*-sheet, random coil, and *β*-turn structure where *β*-sheet is evidently the most intense component. The results showed that *β*-sheet was the dominant component (58.6%), *α*-helix was the least (12%), and the content of *β*-turn and random coil were 15.39% and 14.5%.

## 4. Discussion

Fucoidanase could be isolated from marine invertebrates as *Haliotis* sp., sea cucumber, sponges, and molluscs [34] and was produced by marine bacteria as *Pseudomonas atlanica*, *P. carrageenovora*, *P. alteromonas*, *Vibrio* sp., *Bacillus *sp. HTP2, *Pseudoalteromonas citrea*, Family flavobacteriaceae [19, 24] and marine fungi *Dendryphiella arenaria* TM94 [26], *Fusarium *sp. LD8 [27], *Aspergillus niger, Penicillium purpurogenum*, *Mucor *sp. [28]. 

Fucoidanase had different molecular weight in different organisms. The molecular weight (*M*_*w*_) of LD8 fucoidanase was 64 kDa; it is close to that of fucoidanase E1, E2, and E3 of *Vibrio* sp. N-5 (39 kDa, 68 kDa, and 68 kDa, resp.) [33], while the *M*_*w*_ of LD8 fucoidanase was lower than that of fucoidanase from hepatopancreas of* Patinopecten yessoensis* (100–200 kDa) [14] and *Dendryphiella arenaria* TM94 (180 kDa) [35]. 

Fucoidanase from *Fusarium* sp. LD8 was more sensitive to pH and temperature. The catalytic activity of the fucoidanase of LD8 reached maximum at pH 6.0, which is very close to that of the fucoidanases from bacteria *Vibrio* sp. N-5 and *Dendryphiella arenaria* TM94. The enzyme activity decreased rapidly in LD8 when pH is below or above 6.0. At pH 5.0 and 7.0, the enzyme retained 68.2% and 86.4% of the enzyme activity at pH 6, respectively. The fucoidanase from LD8 had a relatively higher optimal temperature (60°C) compared with that of bacteria *Vibrio* sp. N-5 (37°C), Flavobacteriaceae SW5 (room temperature), and marine fungi *Dendryphiella arenaria* TM94 (50°C). The temperature of half inactivation of LD8 fucoidanase was 50°C, while that of bacteria *Vibrio *sp. N-5 was 65°C [33].

Effects of temperature on the secondary structure of LD8 fucoidanase were studied by Gaussian fitting to the deconvoluted spectra of fucoidanase at amide I region [36, 37]. A decrease of the *β*-turn structure and an increase of *α*-helix of amide I region had been observed when treated temperature was below 60°C. While treated temperature was above 60°C, the contents of *α*-helix,*β*-sheet, random coil, and *β*-turn had no changes. The above result was consistent with our conclusion that the optimal enzyme reaction temperature was 60°C; The enzyme activity decreased rapidly in LD8 below or above pH 6.0. It was suggested that the enzyme activity of fucoidanase was closely related to the proportion of *α*-helix and *β*-turn structure with no direct relation to *β*-sheet and random coil structure.

## Figures and Tables

**Table 1:** Summary of Purification of Fucoidanase.

Purification step	Total protein (mg)	Total activity (IU)	Specific activity (IU*·*mg^−1^)	Purification (fold)	Yield (100%)
Crude enzyme	11668.43	127.96	0.011	1	100
Acetone precipitation	509.23	80.43	0.16	14.5	62.8
Sephadex G-100	120.43	30.64	0.25	22.7	23.9

**Table 2:** Fucoidanase and fucoidanase-related activity of purified protein.

Enzyme activity	Fucoidanase	E2 Fucosidase	Amylase
IU*·*mL^−1^	1.88	—	—

—: no enzyme activity.

**Table 3:** The results of Gaussian fitting of fucoidanase in amide I region.

Position	Area	Content (%)	Components (assignments)
1606	7.89140	21.1	*β*-sheet
1618	7.40320	19.8	*β*-sheet
1631	2.36538	17.7	*β*-sheet
1645	5.40532	14.5	random
1658	4.31620	11.5	*α*-helix
1672	3.38760	9.06	*β*-turn
1685	6.60977	6.33	*β*-turn

## Abbreviations

LMWF: The low molecular weight fucoidan
*K*_*M*_: Michaelis constants
*V*_max _: Maximum reaction velocities
*M*_*w*_: Molecular weight.
